# A simple model of mechanical effects to estimate metabolic cost of human walking

**DOI:** 10.1038/s41598-018-29429-z

**Published:** 2018-07-20

**Authors:** Salman Faraji, Amy R. Wu, Auke J. Ijspeert

**Affiliations:** École Polytechnique Fédérale de Lausanne (EPFL), Lausanne, Switzerland

## Abstract

Since the advent of energy measurement devices, gait experiments have shown that energetic economy has a large influence on human walking behavior. However, few cost models have attempted to capture the major energy components under comprehensive walking conditions. Here we present a simple but unified model that uses walking mechanics to estimate metabolic cost at different speeds and step lengths and for six other biomechanically-relevant gait experiments in literature. This includes at various gait postures (e.g. extra foot lift), anthropometric dimensions (e.g. added mass), and reduced gravity conditions, without the need for parameter tuning to design new gait trajectories. Our results suggest that the metabolic cost of walking can largely be explained by the linear combination of four costs—swing and torso dynamics, center of mass velocity redirection, ground clearance, and body weight support. The overall energetic cost is a tradeoff among these separable components, shaped by how they manifest under different walking conditions.

## Introduction

Energetic economy has been shown to have a large influence on human walking behavior. For example, at a given speed, humans tend to walk with a preferred step length that coincides with minimum metabolic cost^1,2^. Despite the complexity of relating walking mechanics to energetic expenditure, past studies have determined important contributors towards the overall energetic cost of walking, such as the work performed during step-to-step transitions to redirect the center of mass (CoM) velocity and the cost of generating muscular force for body weight support and for leg swing^3–6^. To the best of our knowledge, however, no study to date has used walking mechanics to present a unified cost landscape that can predict metabolic cost under various walking conditions. Without understanding the major energetic contributions, it would be difficult to identify the energetic consequences of compensatory movement in abnormal gait or prescribe effective treatment. Likewise, reducing the metabolic cost of impaired walking towards normative levels may contribute towards the efficacy of prosthetic and orthotic devices^7^.

The metabolic cost of walking is the overall energy consumption from many different mechanisms in the body, including muscle dynamics, blood circulation, and aerobic processes^8^. In human gait experiments, this cost is typically calculated from measurements of oxygen consumption and carbon dioxide production minus the basal metabolic rate of standing to yield net metabolic power^9^. Metabolic cost is conventionally expressed in two different ways: the metabolic energy consumed per unit of time (metabolic rate or power) or the metabolic energy consumed per unit of distance (Cost of Transport, CoT).

Several papers have tried to relate the metabolic cost of walking to walking mechanics. Despite a measurable energetic cost, the average mechanical work per stride during steady-state level walking is near zero. In early biomechanical studies, Saunders identified various geometric determinants of walking (e.g. torso rotation, lateral torso tilt, midstance knee flexion) and postulated that all these determinants are the result of body’s best effort to flatten and smoothen the CoM trajectory, i.e. minimizing accelerations^10^. To the contrary, numerous studies have identified the energetic benefits of non-flat pendular dynamics of the CoM due to potential energy and kinetic energy exchange^11^. These simple inverted pendulum walking models demonstrate that no work is needed during each step. Rather it is the positive work performed to restore lost collisional work during the step-to-step transitions that dictates metabolic cost^3^.

Other mechanisms, such as swing leg dynamics and torso balance, are also important contributors to energetic cost during walking. While leg swing can be explained by passive pendular dynamics^12^ and thus is sometimes unmodeled in simple walking models^13^, experimental studies have shown that it contributes approximately 10% of the net metabolic cost^6^. Despite its relatively small weight, leg swing oscillations forced at frequencies above the natural frequency can have a significant energetic consequence due to muscle force production at the swing hip^5^. Furthermore, due to pendular falling dynamics, the pelvis also has a considerable acceleration in walking direction. This requires the stance hip muscles to apply torques to the torso, which are comparable in magnitude to swing torques^14^.

Modeling the energetic cost of walking is challenging due to the complexities of the musculoskeletal system and its intricate relationship with neural locomotor mechanisms^15^. Translating from chemical processes at the molecular level to muscle force production and metabolic consumption is nontrivial and difficult to measure. The indeterminate relation between muscle work and joint mechanical work further complicates biomechanical analysis and modelling. Despite these difficulties, both sophisticated neuromusculoskeletal models (e.g.^16^) and simple inverted pendulum models (e.g.^17^) have shown that minimizing some form of metabolic cost of transport leads to patterns similar to those of healthy human gait. While the complexity of the former models precludes further insight, the simple walking models permit linear separation of cost factors, such as pendular and swing dynamics^17^. Experimental studies suggest that a large portion of the energetic cost of walking can be attributed to a few factors (e.g. ~28% for body weight support, ~45% for CoM work^4^), but it is still unclear if and how these determinants can be combined to predict metabolic cost under various walking conditions.

We propose a simple metabolic cost model to provide meaningful estimates of the human metabolic rate under general walking conditions. First, to encapsulate walking dynamics in a simple manner, we utilized a 3D linear model that can describe major sagittal plane and frontal plane dynamics (i.e. pendular falling, swing and torso-balancing effects) over a wide range of walking speeds and step frequencies^18^. While that model can capture horizontal energetic contributors during normal walking, it cannot fully capture the empirical CoT data as a function of speed and step frequency from Bertram^1^. Therefore, we needed additional components to capture such unmodeled costs.

We demonstrate that the total energy expended by the body is largely determined by a linear combination of four main mechanical components (i.e. linear separation premise). These are (i) sagittal and frontal dynamics (termed “3LP dynamics”), (ii) CoM vertical velocity redirection, (iii) ground clearance, and (iv) body weight support. We chose these four components because previous studies have shown that they contribute greatly to the metabolic cost of normal walking. They were also the minimum number of components needed to reproduce Bertram’s cost surface, especially at the extreme locations. To scale mechanical work components to metabolic cost, we used both a constant efficiency of 25% and a variable efficiency dependent on step frequency.

After building the model with fits to Bertram’s data, we tested model validity and generality by replicating walking conditions from six other experiments in literature and comparing model predictions with reported energy measurements. The simulated experiments from literature were intended to investigate each cost component. CoM redirection cost was examined with step width studies^19^, and the swing component was evaluated with added mass to the leg^20^. We tested the cost of additional foot lift during swing^21^ to isolate and further assess the ground clearance component. Comparisons with simulated reduced gravity conditions^22^ further evaluated the effect of gravity on the vertical cost components. The data from flat walking experiments^23^ were used to isolate weight support component. Finally, to test how the model fares with varying anatomical properties, we also investigated the metabolic cost of walking for obese individuals^24^.

While these experiments can induce any number of biomechanical changes, we were interested in the choice of step frequency within the experimentally-modified cost landscape. We chose step frequency over other gait parameters because we can use the speed-step length surface of our cost model to estimate the optimal step frequency from the known experimental walking speed. Therefore, the model evaluated the cost landscape to predict the optimal step frequency (with respect to minimizing CoT), without any knowledge of experimental data other than imposed test conditions of walking speed, experimental parameter change, and subject mass and height. We focused specifically on how well the model could predict reported trends by comparing coefficients from a linear or quadratic fit (depending on the original experiment). We also estimated the contributions of each of the four mechanical components and how they change under different walking conditions, insights which cannot be easily obtained from empirical testing.

## Results

**Figure 1:**
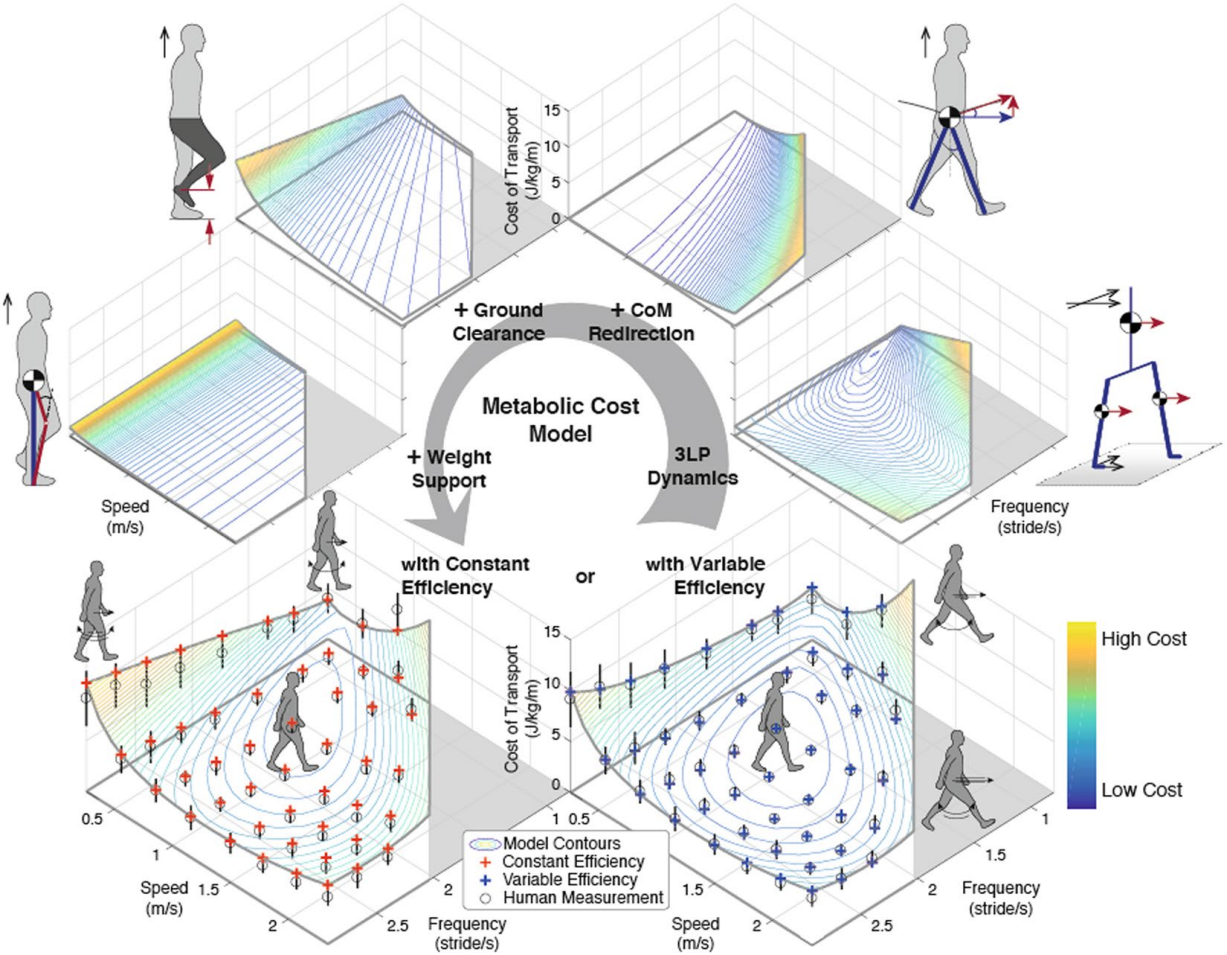
The metabolic cost model and its four components, shown as cost of transport, at different walking speeds and step frequencies with experimental data reported in Bertram^1^ for comparison. The overall cost of transport is composed of the swing and torso cost from sagittal and frontal dynamics (3LP dynamics), CoM velocity redirection, ground clearance, and weight support costs. Each component is dominant at different speed-step frequency combinations. CoM redirection is costly at long step lengths, foot lift at slow speeds and high frequencies, and weight support at slow speeds. These components can be combined with constant muscle efficiency (red crosses) or variable efficiency (blue crosses) to yield costs more similar to experimental data (mean represented by black circles, standard deviation by vertical lines).

Our model produced a metabolic cost surface by the superposition of four components, each with its own energetic penalty profile, over a range of speeds and step frequencies (shown as cost of transport in Fig. 1). We observed from our model that the metabolic cost landscape is composed of energy tradeoffs. Walking at low speeds with high frequencies is penalized by leg lifting effects. Taking larger steps increases costs due to CoM redirection, as was found previously with simulation and empirical data in^3^. Walking at very slow speeds and slow frequencies is costly due to weight support.

We reproduced Bertram’s speed-step frequency study in simulation and compared his measurements with the output of our cost model. As expected due to model fitting, the simulated cost surface corresponded well with empirical data (Fig. 1), and a variable muscle efficiency term provided a better match. Model outcomes were not statistically significantly different from data for constant (*p* = 0.152 ± 0.234, mean ± s.d.) and variable efficiencies (*p* = 0.377 ± 0.273, mean ± s.d.). Model outcomes fell within the data’s 95% confidence interval (47% for constant, 92% for variable efficiency), indicating that our linear separation premise could potentially provide an explanation for the real data. Model’s optimal cost of transport was 2.13 J/kg/m at 1.03 m/s with a step frequency of 1.88 Hz with variable efficiency. Using a constant efficiency yielded an optimal cost of transport of 2.62 J/kg/m (at 0.925 m/s). For both gaits, approximately 50% of the costs were due to ground clearance.

**Figure 2:**
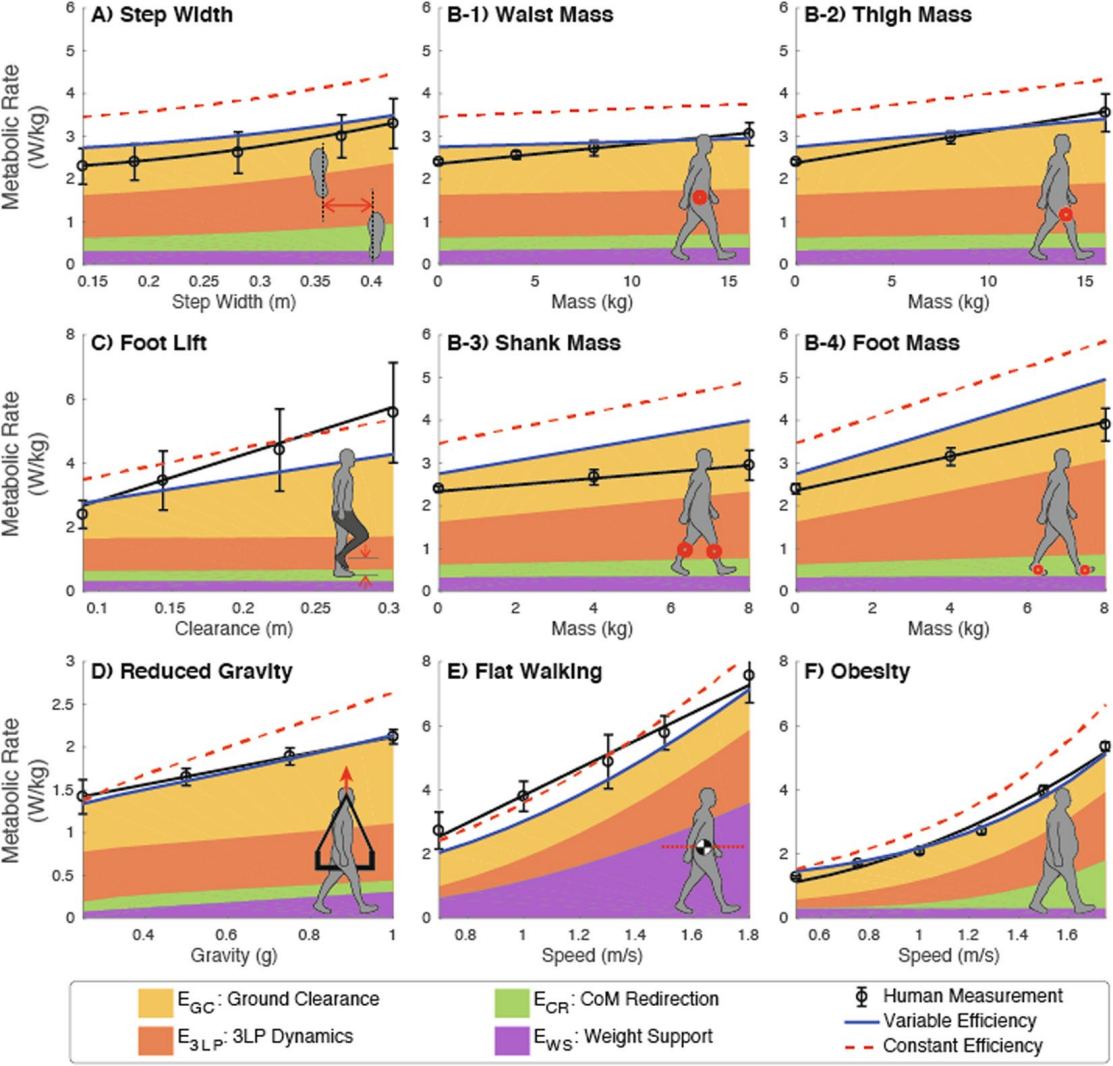
Comparison of model metabolic rate (red lines) with data from six walking experiments from literature (black circles, solid lines). Model predictions include with variable efficiency (solid blue line) and with constant efficiency (dashed red line). The six comparisons were on (**A**) step width^19^, (**B**) added mass to the leg^20^, (**C**) extra foot lift^21^, (**D**) simulated reduced gravity^22^, (**E**) CoM flat-trajectory walking^23^, and (**F**) walking with obesity^24^. Fitting equations, from the original experiments when possible, were used to investigate trends (see Table S1). Patch layers represent the contribution of each cost component (yellow: ground clearance, orange: 3LP dynamics, green: CoM redirection, purple: weight support).

Without changing model parameters (other than the experimental variable), we were able to reproduce experimental data from six different walking conditions and compare model estimates with empirical data (shown in Fig. 2, quantified in Tables 1 and S1). The model generally could estimate changes related to gait configurations (e.g. step width, flat walking) but not other changes (e.g. anatomical, added mass at the shank). The model was also better at estimating trends (metabolic cost as function of the experimental variable) than offsets.

**Table 1:** Trends, offsets, and goodness-of-fit values from linear fitting of correlations between metabolic rates from model estimates and metabolic measurements.

Experimental Parameter (x)	Trend a		Offset b		R^2^	
	Var.	Const.	Var.	Const.	Var.	Const.
Step Width^19^ (m)	0.758	0.998	0.981	1.150	1.000	1.000
Added Mass^20^: waist (kg)	0.278	0. 404	2.097	2.516	1.000	1.000
Added Mass^20^: thigh (kg)	0.547	0.716	1.451	1.763	1.000	1.000
Added Mass^20^: shank (kg)	2.038	2.360	−2.016	−2.053	1.000	1.000
Added Mass^20^: foot (kg)	1.378	1.490	−0.504	−0.045	1.000	1.000
Extra foot lift^21^ (m)	0.497	0.611	1.425	1.858	1.000	1.000
Reduced Gravity^22^ (g)	1.145	1.796	−0.293	−1.157	1.000	0.998
Flat Walking^23^: Flat (m/s)	1.074	1.228	−0.936	−0.959	0.986	0.989
Obesity^24^: Obese (m/s)	0.897	1.250	0.303	0.021	0.989	0.995

For the measurements, we used polynomial equations reported in the original papers since we did not have access to individual subject data. The goodness-of-fit values are close to one, indicating that model estimates follow similar polynomial trends found in the original papers. Both constant efficiency estimates and variable efficiency estimates (abbreviated “Const” and “Var” respectively) are shown. A slope of unity with zero bias means perfect agreement with empirical data (see Fig. S1 for visualization).

### Step width

To examine 3LP’s ability to produce motions in the frontal plane, we compared model predictions against step width experimental data (Fig. 2A). Like the original study, estimated metabolic costs varied quadratically with step width (*R*^2^ = 1.000 for both constant efficiency and variable efficiency). Metabolic cost matched empirical data fairly well in trend (0.758 for variable efficiency and 0.998 for constant efficiency estimates against metabolic data, where 1.000 indicates a good fit) but not in offset (0.981 and 1.150, variable efficiency and constant efficiency). Step width costs were dominated by increases in the 3LP and CoM velocity redirection costs.

### Added mass on the leg

Additional mass was added to the model equivalent of a foot, shank, thigh, and waist to evaluate leg dynamics in the sagittal plane. These added masses led to a linear increase in metabolic cost (*R*^2^ = 1.000). As also observed in the original experiment, added mass was more costly when placed at distal location than proximal ones (Fig. 2B). Added mass to the foot increased metabolic cost the most while adding mass to the waist had the least effect. However, the model also overestimated the distal mass cost increase rates (shank: 2.038 and 2.360, foot: 1.378 and 1.490, variable efficiency and constant efficiency) and underestimated the proximal mass cost increase rates (thigh: 0.547 and 0.716, waist: 0.278 and 0.404, variable efficiency and constant efficiency). Swing dynamics (3LP) and ground clearance played a greater role with distally located added mass than proximal mass placement.

### Extra swing foot lift

We applied various foot lift heights to estimate the metabolic cost of clearing the ground during swing. The model, predicting a linear increase (*R*^2^ = 1.000), underestimated the metabolic cost in trend (0.497 for variable efficiency, 0.611 for constant efficiency) with an offset in magnitude (1.425 and 1.858, variable efficiency and constant efficiency). Not surprisingly, the leg lifting cost accounted for the majority of the increase with some contribution from swing dynamics (Fig. 2C).

### Simulated reduced gravity

We conducted two comparisons to further investigate the cost of body-weight support. For the first study, we investigated simulated reduced gravity, where a counter weight force is applied to the upper-body, not to the entire body as under true reduced gravity conditions. The model estimated a linear increase of energy with gravitational acceleration (*R*^2^ = 1.000 for variable efficiency, *R*^2^ = 0.998 for constant efficiency, Fig. 2D). In trends, the variable efficiency estimate (1.145) provided a better match to empirical data than the constant efficiency (1.796). As gravity decreased, weight support cost reduced as expected^4,22^, along with CoM redirection and ground clearance. 3LP costs also indicated that in very low gravities, leg swing motion becomes costly again.

### Flat-trajectory walking

The second evaluation for weight support cost replicated flat walking (i.e. minimal vertical CoM movement) studies, which demonstrated that reducing COM displacement does not lead to reduced energetic cost^23,25^. Here we used experimental data from Ortega^23^. Here we varied the stance knee angle with simple inverse kinematics as a function of stride (see Methods) to model a constant CoM height trajectory. The cost of flat walking increased linearly with speed (*R*^2^ = 0.986 for variable efficiency, *R*^2^ = 0.989 for constant efficiency, Fig. 2E). The cost model estimated the main trend reasonably well (1.074 for variable efficiency, 1.228 for constant efficiency) with some offsets (−0.936 and −0.959, variable efficiency and constant efficiency). We observed a substantial increase in weight support cost. Thus flat walking creates unfavorable muscle-related changes, which agrees with reports of increased muscle activation and co-contraction during flat walking^26^.

### Walking with obesity

Finally, to investigate changing anatomical properties, we estimated the metabolic cost of walking for obese individuals. We did not expect to be able to reproduce this condition due to the simple scaling of body mass in our model. We found that this cost increased quadratically with speed (*R*^2^ = 0.989 for variable efficiency, *R*^2^ = 0.995 for constant efficiency, Fig. 2F) but overestimated trends (0.897 for variable efficiency, 1.250 for constant efficiency). The majority of the cost increases were due to CoM redirection and 3LP dynamics.

## Discussion

We sought a simple but unified model that could predict the metabolic cost of walking at various speed and step frequency combinations, as well as be generalizable to a range of different walking situations and anthropometric dimensions. We proposed that this could be achieved with a linear combination of four main components: the costs of swing and torso dynamics, CoM velocity redirection, ground clearance, and weight support. To test the model’s linear separation premise, we used a combination of these components to reproduce empirical speed-step frequency data and then tested the model with six different experiments. Overall, this simple model was able to predict some of the energetic trends and magnitudes reported in biomechanically important experiments, demonstrating that linear combinations of these four components could constitute the main metabolic determinants of walking.

Model composition suggests that the optimal metabolic cost is moderated by tradeoffs among different component surfaces. This is comparable to the tradeoffs^1,17^ producing the optimal speed-step frequency curve found in humans^27,28^. As other studies have found, CoM redirection costs penalize longer step lengths^3^, and swing costs penalize fast step frequencies^5^. The model also suggests that weight support costs are more prominent at slow walking speeds, and increasing the frequency at slow speeds incurs high ground clearance costs due to leg lifting.

Our model estimates were similar to those of other energetic models and human studies aimed at decoupling the cost of walking. At the optimal (preferred) speed and step frequency, we found that 24% of the optimal cost can be attributed to swing and 76% to stance. In comparison with computational approaches, neuromuscular models with muscle models also estimated approximately 30% for swing and 70% for stance^29^. We found that swing costs increased with greater step frequency, to the contrary of Umberger’s model^29^ but in agreement with experimental results from Doke^5^.

The cost model was able to estimate energetic cost under various speed-step frequency combinations in both trend and magnitude. The model only differed at very large step lengths, where the linear decoupling of horizontal and vertical motions is weaker, due to larger CoM vertical excursions. The relative accurate predictions elsewhere imply that the four cost landscapes do encompass the overall energetics (Fig. 1) and thus can be used to yield further insight. Our dimensionless cost of transport (energy over body weight and distance) at the model’s preferred walking speed (1.03 m/s) and step frequency (1.88 Hz) was 0.217, a difference of 8.7% in comparison to the average experimental value of 0.2 using net metabolic cost at 1.25 m/s^21^. More physiological models and energy calculations had similar error magnitudes. In comparison with net cost, Endo and Herr’s model had ~10% error^30^. Compared with human gross cost of transport of 0.3^21^, sagittal plane walking models from Umberger *et al*.^31^ and Song and Geyer^32^, who used the same muscle energy formulations, had approximately 15% error and 5% error, respectively. Our model did not greatly overestimate costs like other 3D models (e.g. by 63%^16^), and Roberts *et al*.^33^, who included measured kinematics and kinetics, had a 12% error.

We tested if the model could estimate metabolic cost of six experiments without knowledge of the step parameters chosen by subjects. Using only the reported subject mass, subject height, experimentally-fixed walking speed, and experimental variable, the model estimated an optimal step frequency (Fig. S2) which was very close to the measured frequency in most of the six experiments. Interestingly, despite large differences between predicted and empirical step frequencies for the reduced gravity and flat walking condition, model cost estimates are not very different from measured costs, indicating that perhaps the model is not sensitive to the choice of step frequency.

Since the model was fit to Bertram’s CoT surface, we expected our model to be better at estimating the energetic consequences of changing speed and/or frequency. This is partially reflected in the flat walking and obesity experiments, where walking speed was the main experimental variable (see Fig. 2). Model predictions in flat walking experiment were reasonable, likely also due to the use of the Alexander-Minetti curve for the weight-support component. Predictions in increased step width and reduced gravity conditions were also relatively good, perhaps because energetic changes were small, and the model roughly stayed within linear regions. Of the six experiments, we did not expect the model to estimate the metabolic cost of obese walking. While the model obtained a decent fit, the obesity prediction was similar to those for normal walking. The model cannot differentiate well between obese and normal walking.

Added mass and extra foot lift experiments extend beyond linearity and decoupling assumptions, which may explain why model predictions failed. The human knee-ankle mechanism is also much more complex than in our model. We can attribute some of the estimation errors to the efficiency of muscles. For example, using a lower walking efficiency of 19.5% improves trend estimates for foot lift (Fig. S3). However, with the 3LP component, it is unclear whether the errors are due to decoupling assumptions, muscle efficiencies, or some other unknown parameter that we did not consider.

We found that prediction trends were relatively insensitive to the choice of free parameters—mid-stance knee angle *θ*, max heel lift height *c*, muscle efficiency *η*, and center of pressure CoP (Fig. S3). Except for reduced gravity and flat walking, changes in knee angle mainly changed biases and not trends. Heel lift and center of pressure variations produced minor changes in cost estimation. Muscle efficiency seemed to have the largest influence, affecting the trend for normal subjects and obese subjects. Therefore our cost model is more robust to parameter variations in predicting trends, but less precise when estimating exact magnitudes. More accurate trends are arguably more important than magnitudes when estimating human energetic consumption because relative changes between nominal and new conditions can be detected.

Our cost model successfully demonstrates the speed-constrained optimization hypothesis (see Fig. S2) proposed by Bertram^1^ as well as provides reasonable trend estimates of human energy expenditure under varying anatomy and walking conditions. The intrinsic power of this method comes from the separation principle—the decoupling of different phenomena in a linear fashion and studying each separately. Linear separation of energy-related components is not uncommon, as evidenced by both simple models (e.g. summation of push-off, hip actuation, swing leg costs in Kuo^17^) and more complicated musculoskeletal models (e.g. addition of various heat rates and mechanical work in Anderson^16^). Linear separation implies more than an addition of energy costs, suggesting that the decoupling of highly complex and interconnected human locomotor functions could still encompass the major costs of walking. As is evident from the added mass experiment and extra lifting conditions, the vertical and sagittal dynamics can, to some extent, be separated. This may be similar to how the control of walking in the sagittal plane and the frontal plane can be considered separately, as demonstrated in simulations^18,34^ and in human experiments^35^. Similarly, the separation of stance, swing, and balance control, with limited sensory exchange despite their inherent interconnectivity, has been shown to simplify gait coordination on robots and assistive wearable devices^36^.

The six validation experiments were chosen to isolate cost components. For example, foot lift experiments were to study foot-to-ground clearance and reduced gravity to study weight support. The swing cost in 3LP could also be isolated to some extent by the addition of distal masses to the legs. Due to gait geometry, however, vertical CoM redirection cost highly correlates with the horizontal falling dynamics in 3LP. Comparisons with the flat walking experiment attempted to cancel the vertical component but significantly increased the weight support cost. Additional experiments and analysis are needed to further separate each cost component. For example, a modified flat walking experiment with some weight support could possibly isolate the 3LP cost. We can further challenge 3LP and CoM redirection costs by investigating asymmetric walking gaits (e.g. on inclined terrains, with constant pulling forces or with extra torso bending). We can also investigate lateral swing dynamics in 3LP with swing foot circumduction experiments. These extra validations may require extensions of 3LP or experiments with human subjects, which we consider for future work.

Our use of mechanical measures to estimate metabolic cost is limited by their rather abstruse relationship. Metabolic cost can be incurred without net mechanical energy, such as during cyclic locomotion or muscle co-contraction. Observed mechanical work at a single joint could entail not only positive muscular work, but also contributions from elastic tendons or bi-articular muscles, which act across multiple joints. Positive and negative muscle work also contribute differently towards metabolic cost^37^. Thus it is unsurprising that the ratio of whole body metabolic cost to mechanical work can vary widely depending on walking condition^26,38^. Nonetheless, simple mechanical models and biomechanical experiments have shown that mechanical measures could largely account for changes in metabolic cost^3,39^.

We were positively surprised by favorable energetic trend predictions. Not surprisingly, there were also inaccuracies in estimated magnitudes. Multi-segment leg motions and internal muscle properties were highly simplified. Telescoping actuators, meant to reproduce knee-ankle energy pumping and absorption mechanisms, are not physiological but can produce human-like pendular dynamics in the sagittal plane. Passive coupling between knee flexion and leg swing is also missing, which implies that the hip actuator contributes more to swing the leg. Unmodeled changes in leg inertia during knee flexion could also explain trend differences for the added shank mass and extra foot lift experiments. Indeed, we have omitted several features of human walking, including a non-infinitesimal double-support phase, a non-constant muscular efficiency, arm and transversal pelvic motions, and more anthropometric features. For example, while 3LP does include the mechanical work to balance the torso, our simplified upper body model neglects upper body angular momentum with no arms and a torso that remains vertical with respect to gravity. We believe these missing features do contribute to the observed differences between estimated and empirical data in some of the experiments, especially the use of muscular force to regulate whole body angular momentum^40,41^. Arm dynamics, for instance, affect metabolic cost rates, with an increase of 26% if swinging anti-normally^42^.

More complex (e.g. nonlinear) or physiologically complete models (e.g. neuromuscular, multi-segmental model) could provide more realistic predictions in different walking conditions. For example, this model uses whole body mechanical work to capture the work performed by the muscles, instead of summing energy consumptions at the muscle level^43^. Accounting for muscle dynamics could provide better estimates and predict muscle-related effects that our model cannot capture. Additional features such as muscle co-contraction and realistic mass distribution could provide a better energy estimate in experiments, such as obesity, but might overfit the general CoT surface. For instance, Sasaki *et al*.^44^ found that the error between joint work and total musculotendon work could be as high as 7%, which could affect our mechanical work-derived costs. This may also account for our model’s underestimation of cost for the flat walking experiment. More complicated models may also require potentially time-consuming optimization routines contending with more tuning parameters and appropriate objective functions to find periodic gaits.

Here we have proposed a minimalistic model to capture main trends in the CoT curve. The proposed cost model is based on a linear walking model, for which periodic gaits can be easily found. Such computational advantage makes our model suitable for prediction of transient walking conditions, such as accelerations and decelerations in walking speed. The proposed cost model can also be easily tailored to subject height, leg length and pelvis width. Its effectiveness in estimating changes with body weight (due to obesity results) requires further investigation. By decomposing the overall cost landscape into different components, our model suggests the dominant physical effects of different walking conditions. Quantifying these components for some gait condition can be performed empirically and would require clever but possibly laborious experimental procedures and apparatuses. Here separate experiments are not needed to study the effect of each component. While we acknowledge the difficulty in translating additional gait assistance to reduction of metabolic cost, the resulting decomposition could still help physiotherapists or biomechanists improve assistance or promote rehabilitation by targeting components that contribute the most towards whole body measurements, such as metabolic power. Thus this simple cost model, which can explain a wide range of unusual experiments and their underlying cost contributors, creates insights not easily obtainable in human experiments and potentially valuable towards improving or augmenting human performance.

## Methods

**Figure 3:**
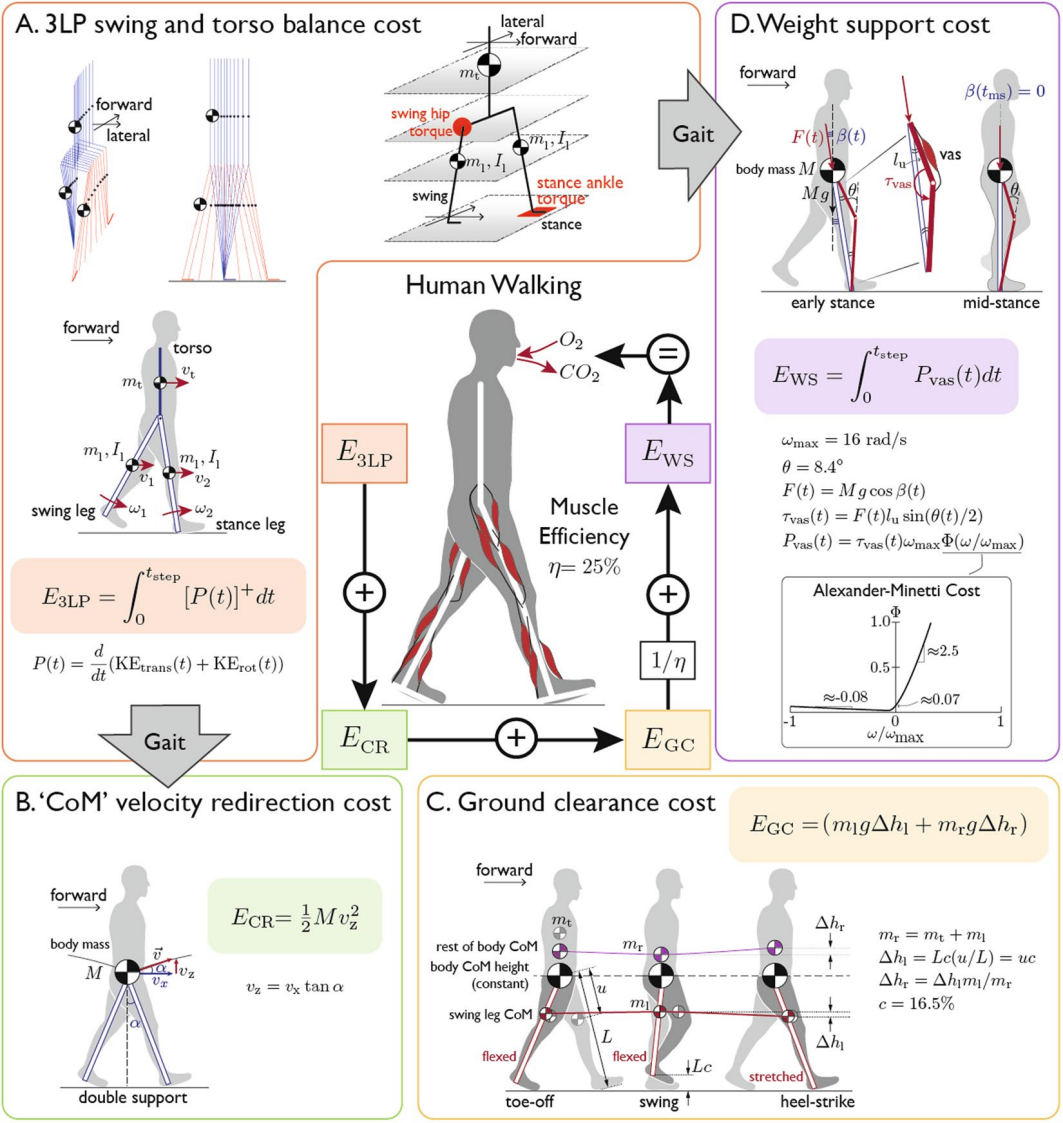
The four energy components of the metabolic cost model and their formulations. The cost components are from (**A**) 3LP dynamics, (**B**) CoM velocity redirection, (**C**) ground clearance, and (**D**) weight support. (**A**) 3LP is composed of three linear pendulums (blue), two represent the legs and one for the trunk. Translational and rotational kinetic energies are calculated from the linear and angular velocities of each segment. The 3LP cost is the integral of the positive component of the kinetic energy change rate. (**B**) CoM velocity redirection cost accounts for the vertical work to change CoM velocity at the step-to-step transitions, which is not accounted for by the 3LP model. Similar to Kuo^48^, the magnitude of the velocity redirection, and thus kinetic energy, depends on geometry (i.e. the angle ***α*** between the legs). This angle comes from 3LP geometry (represented in blue). (**C**) Ground clearance cost is the potential energy to lift the leg. We used a constant ***c*** of 16.5% of leg length for lift height. Since the vertical CoM displacement must be constant, there is a corresponding penalty to move the ‘rest of body’ mass down. (**D**) Supporting the body during stance requires extensor muscular force to keep the leg from collapsing. The metabolic cost of the vasti muscles performing leg extension is calculated from the Alexander-Minetti curve^52^, following the work of Srinivasan^51^. The leg angle ***β***(***t***) is derived from 3LP geometry (blue), and we used a constant knee angle ***θ*** of 8.4 degrees.

Our cost model is composed of four mechanical components: sagittal and frontal dynamics, CoM velocity redirection, ground clearance, and body weight support (see equations and schematics in Fig. 3, cost curves in Fig. 1). The overall metabolic cost is composed of the energies of these mechanical effects, scaled by the inverse of muscle efficiencies (Equation 5). We evaluate our cost model by comparing model predictions with experimental measurements from different walking conditions in literature (Fig. 2).

### Metabolic cost model

#### Model development and choice of components

The four components were determined as follows. We started with the 3LP model, our first component, which was developed in an earlier work^18^. As a great simplification of human dynamics, we did not expect 3LP alone to be able to predict the empirical CoT, and we used Bertram’s cost surface to conceptually help us identify most important components missing in 3LP. One advantage is that 3LP can describe pendular and swing dynamics together. This combination can naturally explain the trade-off between push-off and swing costs^17^ but seems insufficient to explain the overall cost of transport surface in different walking conditions^1^. In particular, 3LP does not account for changing vertical dynamics with large step lengths and does not demonstrate high CoT at slow speeds, as found in humans.

This suggests that the model may be missing cost components, especially in the vertical direction, and we strived to add a minimal number of components to produce the cost surface. Vertical CoM excursions and the associated push-off cost seemed most relevant for the cost of greater step length. Leg lift could possibly explain the high CoT in slow speeds and less dynamic walking conditions^45^. The constant non-zero knee angle reported by^46^ at different walking speeds, which could be related to the significant contribution of weight support on energetic cost^4^, was not captured in any of the previous three components. Therefore, we added a fourth component to our cost model for body weight support. This component captures the metabolic cost of producing an isometric force at non-fully stretched knee angles. At very slow speed and frequency conditions, where the first three components together underestimates Bertram’s CoT surface, this weight support component could explain the mismatch. We used both a constant efficiency and a variable efficiency^26^ to scale mechanical power to metabolic power.

The free model parameters are minimum stance knee angle, amount of ground clearance, center of pressure (CoP) profile, and muscle efficiency. Values for all four were determined by calculating the best fit to Bertram’s data, within the constraint of existing data (see Fig. S3). Best fit was determined by the smallest average p-value between model and human CoT across the entire cost surface. We found that constant values in the middle of reported ranges generally provided a good fit and thus used these values. However, we also found that a variable efficiency parameter had the best fit, especially as a function of frequency. We decided to show estimates for both constant efficiency and variable efficiency to demonstrate the effects of added complexity.

#### 3LP dynamics: swing cost, torso balance cost

To generate gait as well as measure the swing and torso balancing dynamics, we needed a dynamic walking model that can describe active torques at the hip. We used a previously developed mechanical model called 3LP^18^, a linear 3D model composed of three pendulums (one per leg and one for the upper body). Each pendulum has a mass, and each leg also has inertia. Masses, segment lengths, and proportions were taken from human data^47^. The 3LP model also has a pelvis width to produce lateral behavior. To maintain model linearity, all masses and the pelvis remain at a constant vertical height, presuming there are prismatic actuators in the legs that can realize this simplification. The upper body does not tilt or roll, but the leg pendulums can rotate along the sagittal and frontal plane to encode swing or stance motions. The 3LP model is able to simulate torques in swing and stance hip joint as well as center of pressure modulations^18^.

Due to its linear properties, finding walking gaits with 3LP is computationally easy. Given gait frequency and speed as inputs, 3LP prescribes swing hip and stance ankle torques to create the desired motion, enabling simulation of different walking frequencies and speeds. These torques are parametrized with a piecewise linear profile in lateral or sagittal directions separately. Based on the fixed given gait frequency, we form transition matrices (as a function of time) and multiply them together to find a single matrix where gait variables are lying in the null space of this matrix. These variables are initial pelvis and feet positions and velocities as well as parameters for the torque profiles. Then, based on the given gait speed, we can find a unique gait by combining eigenvectors of this matrix where we consider minimizing joint torques to resolve redundancy. The lateral motion of the model is not dictated and instead emerges from this process together with torque profiles that qualitatively look very similar to human profiles^18^. Each step in 3LP is composed of a swing phase where the foot velocity becomes zero at the end, preceded by an infinitesimally short double support phase. One can impose a desired step-width as well, since an infinite number of gaits exist in the linear system of equations. The 3LP swing and torso balance cost is the positive component of overall mechanical power over one simulated step of the 3LP model. Given step time *T*_step_ and translational and rotational kinetic energy KE_trans_ and KE_rot_, the 3LP cost is calculated by:

1 $${E}_{{\rm{3LP}}}={\int }_{0}^{{T}_{{\rm{step}}}}{[\frac{d}{dt}({{\rm{KE}}}_{{\rm{trans}}}+{{\rm{KE}}}_{{\rm{rot}}})]}^{+}dt$$

This integration is actually done for variations of translational and rotational kinetic energies in the sagittal and lateral directions separately. There is no variation in the potential energy due to constant mass heights. The cost of all vertical motions is captured in the other three cost components.

#### CoM redirection cost

An immediate mechanical effect missing in 3LP is the consequences of pendular falling dynamics in the vertical direction. At the end of each step, negative work is performed to redirect the body CoM velocity, and positive work must be performed to recover collisional losses. Both in simulation and in human experiments, the ideal time is to provide a push-off force by the trailing leg right before collision^48,49^. To address the vertical component of this push-off cost, we introduced the velocity redirection work, calculated using 3LP gait geometry. Considering the position of the model’s legs at push-off and the pelvis horizontal velocity vector, we calculated an augmented 3D pelvis velocity vector, orthogonal to the leading leg. The energetic consequence *E*_*CR*_ was then calculated similarly to the method proposed in Kuo^48^ but using the vertical component of the velocity. Assuming horizontal pelvis velocity *v*_*x*_ at the push-off moment and attack angle *α*, both given by 3LP gait, the vertical CoM velocity change due to collisional impact is given by ${v}_{z}={v}_{x}\,\tan \,\alpha$. The push-off work needed to compensate for this loss is:

2 $${E}_{{\rm{CR}}}=\frac{1}{2}M{v}_{z}^{2}$$

We used body mass *M* at the pelvis as a proxy for CoM. Only the vertical component was considered because the forward and lateral costs have already been included in the 3LP model.

#### Ground clearance cost

During the swing phase, humans nominally walk with a nonzero amount of leg lift, possibly to avoid foot scuffing or obstacles. The maximum toe lift over the entire swing phase is few centimeters^50^, whereas during the swing initiation, the heel is already lifted due to the action of rolling on the toes and flexing the knee. Although an extra passive shank lift (due to the leg inertia) happens shortly after push-off, it does not increase the maximum heel height significantly^45^. We assumed prismatic legs which simplify the complex knee mechanism but provide a good approximation of leg CoM trajectories (Fig. 3).

The cost of foot lift is partly attributable to mechanical work, which increases with lift height^21^. Therefore, to associate a cost to foot lift, we simply considered the mechanical work to lift the heel to a fixed maximum height *c* of 16.5% of leg length, the middle of the range of reported maximum heel lift heights over different speeds^45^ (Fig. S3–2). Based on average anatomical data^47^, we calculated the vertical displacement of the leg’s CoM accordingly and associated a potential energy cost which should be provided by the leg muscles. Assuming a heel lift of *c* (as percentage of leg length *L*), a mass *m*_*l*_ for each leg, and a leg CoM located *u* units below the hip joint, the leg CoM lifts Δ*h*_*l*_ = *Lc*(*u*/*L*) = *uc* units with a heel lift of *c*. The energetic consequence is:

3 $${E}_{{\rm{GC}}}=2{m}_{l}{\rm{\Delta }}{h}_{l}g$$

where the gravity is denoted by *g*.

While leg swing encompasses a cost to both swing the leg and clear the ground, 3LP already encodes the swing cost. The ground clearance cost simply accounts for the work to lift the leg vertically. Also note that the lift of the swing leg displaces the body CoM vertically, but vertical motions of the CoM were already considered in the previous cost components. We assume that during the lifting of the swing leg, the rest of the body moves in the opposite direction, in order to keep the CoM at the same vertical level and to avoid interference between cost components. Therefore, the cost *E*_GC_ is the sum of the mechanical work to lift the foot first and then, to lift the rest of the body back to the initial CoM height.

#### Weight support cost

During the stance phase of walking, leg extensor muscles must act to prevent the stance leg from collapsing under the weight of the body. This cost is not captured in the 3LP model and indeed not straightforward to calculate directly based on a mechanical work, especially since muscles are not ideal actuators and consume energy when applying forces isometrically. As a simple model of weight support, we calculated this cost from the knee torque required to maintain a constant knee angle *θ* of 8.4 degrees (Fig. S3–1). This angle was derived from the minimum knee angle at mid-stance, which we observed to be relatively constant over a range of walking speeds^46^. Calculated in a similar manner as Srinivasan^51^, we converted the knee torque to an isometric muscle force applied by vasti group muscles and then calculated the metabolic cost *E*_WS_ from this force production using muscle-specific parameters and Alexander-Minetti metabolic rate curves^52^ (see Fig. 3). Assuming thigh length *l*_*u*_, body mass *M*, gravity *g*, and stance leg angle with respect to gravity *β*(*t*) (determined by 3LP gait geometry, see Fig. 3), the torque required in the knee is approximated by ${\tau }_{{\rm{vas}}}(t)=Mg\,\cos (\beta (t)){l}_{u}\,\sin (\frac{\theta (t)}{2})$, where *θ*(*t*) = 8.4° is the knee angle assumed to be constant in the model. Given vasti group muscles’ maximum rotational velocity *ω*_max_ and the Alexander-Minetti’s cost curve ${\rm{\Phi }}(\frac{\omega }{{\omega }_{{\rm{\max }}}})$, where *ω*(*t*) is the time derivative of *θ*(*t*), the weight support cost is calculated as:

4 $${E}_{{\rm{WS}}}={\int }_{0}^{{T}_{{\rm{step}}}}{\tau }_{{\rm{vas}}}({\rm{t}}){\omega }_{{\rm{\max }}}{\rm{\Phi }}(\frac{\omega }{{\omega }_{{\rm{\max }}}})$$

This simplified method neglects co-activation of the antagonist hamstring muscles during early stance. While previous simulations have found that hamstring muscles contribute little to support the body^53^ (mostly as hip extensors), the cost of exerting muscle forces still contributes to increased metabolic cost due to co-contraction at the knee. More precisely, the cost of net knee torque needed to support the weight may underestimate the summation of individual vasti group and hamstring costs up to 7%^44^.

#### Total cost: scaled by muscle efficiencies

We propose that the overall energetic cost can be approximated by the sum of all aforementioned costs. While the fourth cost *E*_WS_ already accounts for the conversion from mechanical energy to metabolic cost, the first three costs are expressed as mechanical work and need to be scaled by muscle efficiency *η* to be converted from positive mechanical work to metabolic input.

5 $${E}_{{\rm{walking}}}=({E}_{{\rm{3LP}}}+{E}_{{\rm{CR}}}+{E}_{{\rm{GC}}})/\eta +{E}_{{\rm{WS}}}$$

For whole body behavior, muscle efficiency parameter *η* could vary widely depending on walking conditions (e.g. from approximately 20% to 33% at different speeds, (Fig. S3–3 ^26^). We chose to apply *η* = 25%, derived from isolated muscle^54^ and inclined walking^37^ studies and typically used in biomechanics studies^3,4^.

#### Variable muscle efficiency

Inverse dynamics calculations on recorded kinematic data of subjects walking at different speeds result in a variable overall muscle efficiency (Fig. S3–2) when compared to actual oxygen measurements^26^. Since walking frequencies were not originally reported in^26^, we obtained them using the speed-frequency relations reported in^55^. A variable efficiency function was then defined by interpolating muscle efficiencies reported by Massaad as a function of these frequencies. We recalculated the cost model (see Fig. 1) by this variable efficiency function and observed a better match in different regions of the speed-frequency CoT surface, especially in normal walking conditions. Using speed as interpolation variable for efficiency instead of frequency only worked around the optimal walking regions, but perturbed the surface completely in other regions (see Fig. 1).

### Experiment Replication

Six experimental conditions were replicated in simulation with little modifications to the model. Step width^19^, added mass^20^, extra foot lift^21^, and obesity^24^ experiments were recreated by simply imposing the specific parameter varied in the study (step width, additional segment mass, foot lift height, and body mass, respectively). Simulated reduced gravity conditions^22^ was imposed by applying a constant upward force to the 3LP model and scaling half of the ground clearance and weight support costs by gravity reduction factor (see Equations 3 and 4). Note that the leg experiences full gravity (to be comparable with the actual experiment) while the other half of the body is vertically moving in reduced gravity conditions, when calculating the ground clearance cost for this particular case. For all experiments, the model was scaled by the average body mass and height of subjects participating in the experiments, and gaits were found at the experimentally imposed walking speeds.

In flat walking condition^23^, the CoM height was kept constant which, imposed by the constant CoM height trajectory of 3LP, required the knee angle to change with time. To calculate this knee angle trajectory, we solved a simple inverse kinematic problem between the fixed stance foot point on the ground and the pelvis location at each instance of time in 3LP. We superposed a 2-segment leg model composed of thigh and shank segments only. Since the pelvis height is constant in 3LP, these two segments can capture the peak knee angle difference between flat and normal walking conditions during stance phase^23^. Weight support cost was simply calculated by considering the force required for the new knee profile (see Equation 4).

### Analysis

To evaluate the speed-step frequency predictions, we used a paired t-test to determine if the mean of the cost of transport at a given speed and step frequency pair was not statistically significantly different from the model’s prediction (significance defined as p < 0.05). We estimated the cost of transport for each subject from Bertram’s study using subject mass and height. The average and standard deviation of the p-values over all speed and frequency conditions are reported. Since p ≥ 0.05 does not indicate similarity, we also calculated the 95% confidence interval at each reported speed-step frequency and evaluated whether the model prediction was contained within this interval. For the other scenarios, we did not have access to individual subject data and therefore could not perform similar statistical tests. To assess the model under these conditions, we performed a linear fit of model estimates against measured data to determine how well they correlated. A trend of unity represented perfect agreement. Additionally, we fit a linear or quadratic curve to our model estimates, depending on the original fitting equation used in the respective papers (Table S1). We then compared the trend and offset values with those reported.

Given experimental condition and walking speed, the estimated metabolic cost was the minimum with respect to step frequency. This limits model predictions to walking conditions that do not overtly induce strong preferences for other objectives, such as safety or robustness. Model estimates (Fig. 2) and optimal frequencies (Fig. S1) are compared with the reported frequencies. Given the cost surface for different speeds and frequencies, optimal trends for speed constrained, frequency constrained and step-length constrained walking conditions^1^ (see Fig. S2) were determined.

We also evaluated the sensitivity of our results to the model’s four free parameters (mid-stance knee angle *θ*^46^, max heel lift height *c*^45^, muscle efficiency *η*^26^, and center of pressure distance CoP^56^), which were derived from existing measurements of human data (Fig. S3). We repeated the replicated experiments using the minimum and maximum values reported for those parameters, instead of the average, and compared the modified variable efficiency predictions.

## Electronic supplementary material

Supplementary figures and tables
