# Supplementary material for: A simple model of mechanical effects to estimate metabolic cost of human walking
Source: Sci Rep. 2018 Jul 20;8:10998. doi: 10.1038/s41598-018-29429-z (PMC6054663; doi:10.1038/s41598-018-29429-z)
Supplement: Supplementary file 1 — Supplementary figures and tables [file 41598_2018_29429_MOESM1_ESM.pdf]

A simple model of mechanical effects to estimate metabolic cost of human walking

Salman Faraji[1]*, Amy R. Wu[1], Auke J. Ijspeert[1]

[1]École Polytechnique Fédérale de Lausanne (EPFL), Lausanne, Switzerland.
*Correspondence to: salman.faraji@epfl.ch

Supplementary Information:

- Figure S 1
- Figure S 2
- Figure S 3
- Table S 1

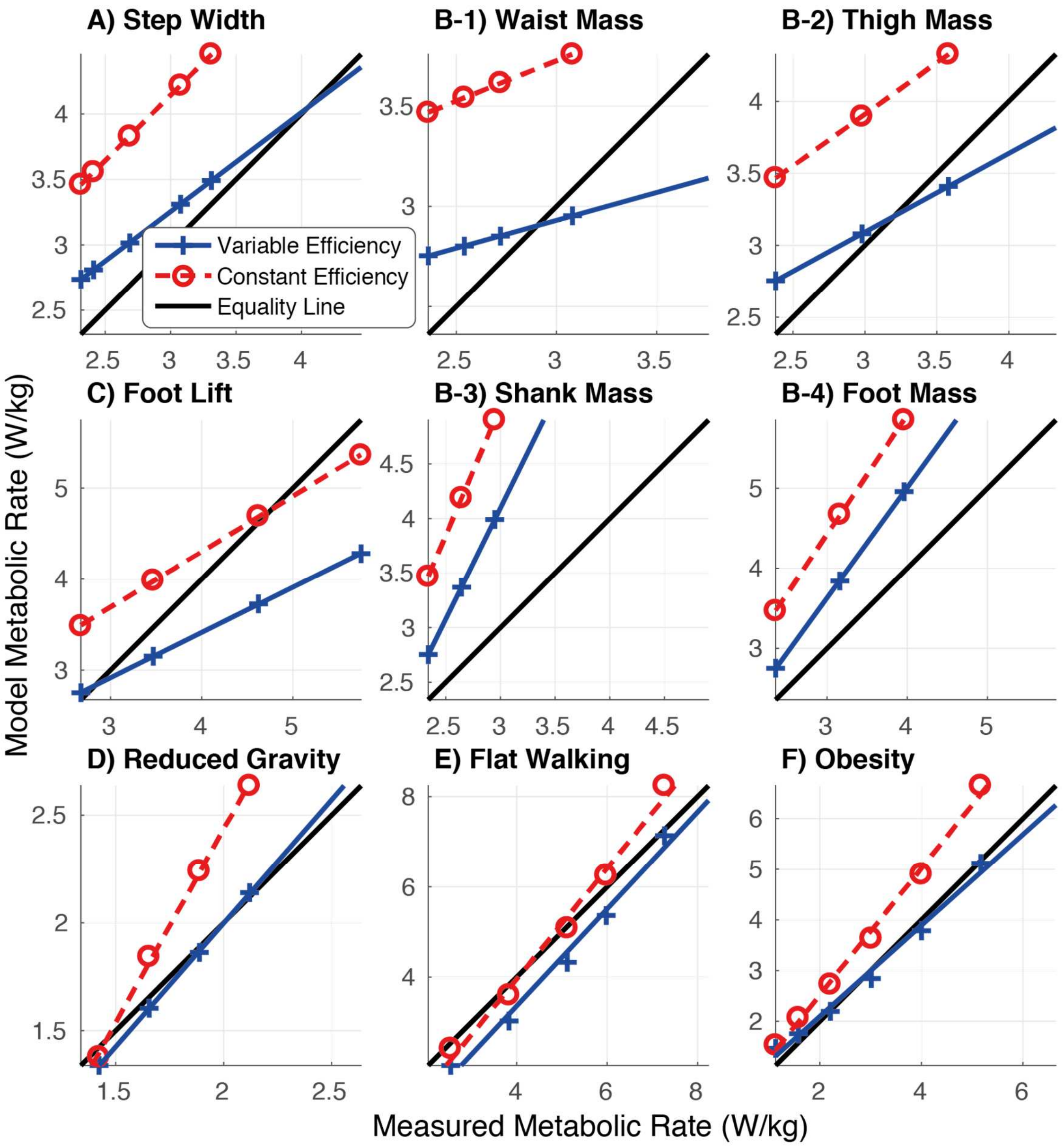

Figure S 1. Correlation between metabolic rates from model estimates and actual measurements for the six experiments under study. Model estimates (blue crosses for variable efficiency, red circles for constant efficiency) are plotted against empirical data, along with corresponding linear fits (solid blue for variable efficiency, dashed red for constant efficiency, see Table 1 for fit values). A slope of unity with zero bias means perfect agreement with empirical data.

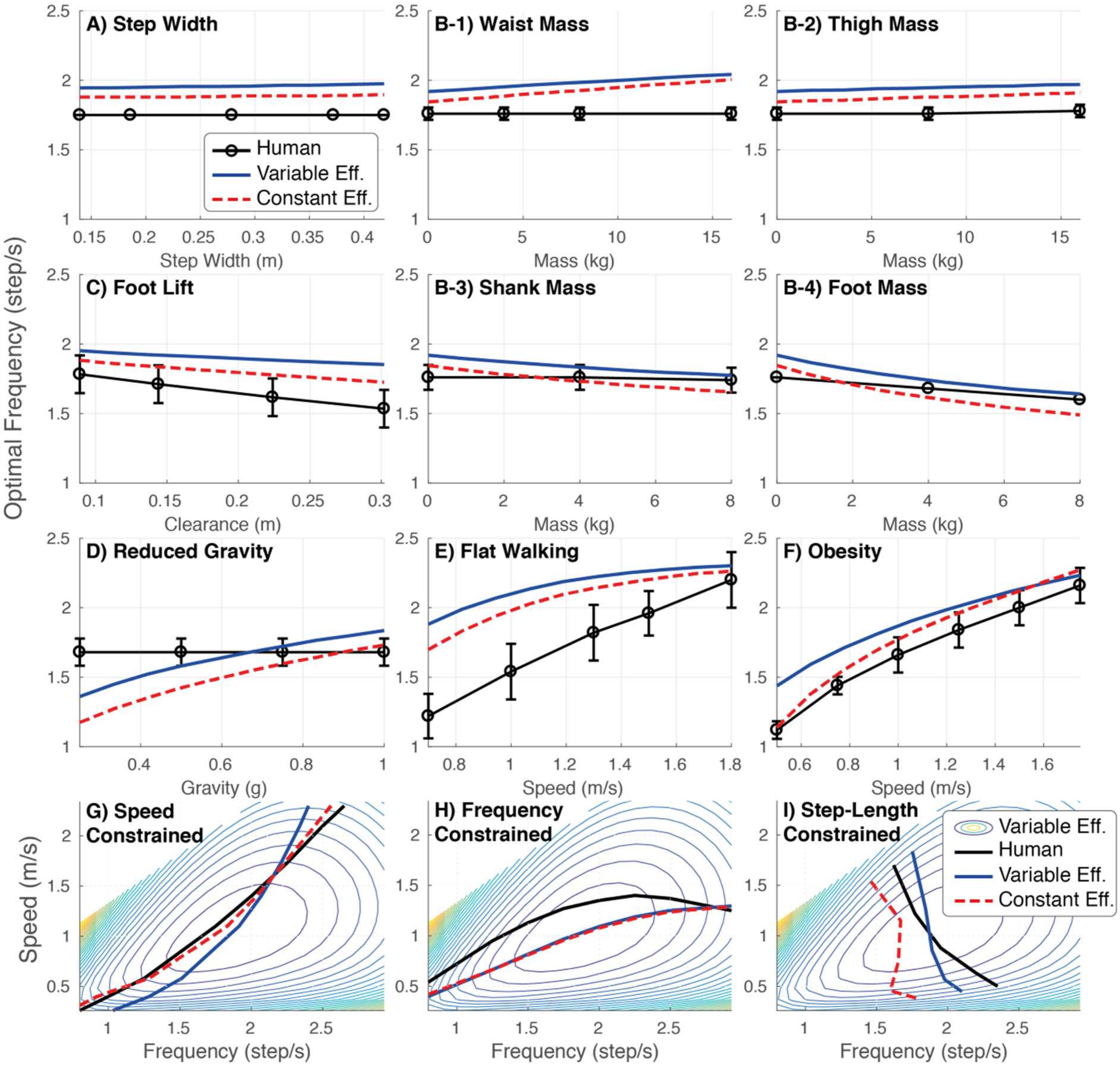

Figure S 2. (A-F) Comparison of experimental step frequencies and model-optimal frequencies by minimizing the cost of transport for each experiment and (G-I) predicted optimal trends by constraining different walking parameters[1]. We repeated optimizations for both constant and variable efficiencies. Increasing and decreasing trends are captured in all conditions despite a bias between the curves. The constant efficiency yields optimal frequencies typically closer to human. The variable efficiency seems unreliable, since there is no evidence for how it actually changes in different experimental conditions. Our model fails at predicting human frequency behavior in reduced gravity, flat walking, frequency-constrained and step-length constrained conditions. We consider muscle efficiencies to be the main reason behind such discrepancy.

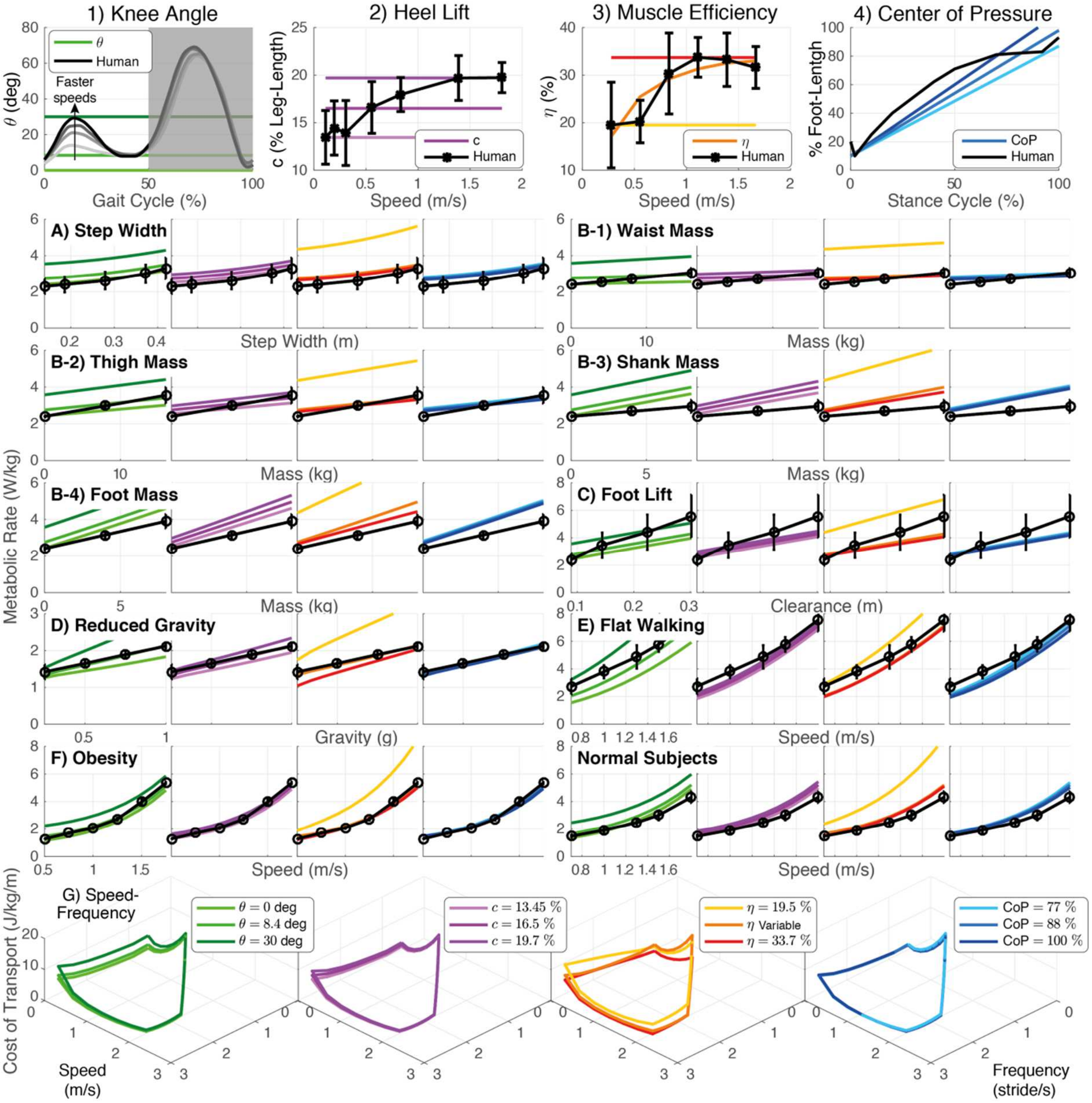

Figure S 3. The choice of free parameters in our model based on human measurements and the sensitivity of the variable efficiency results to parameter changes. (Top Row) Model parameters were based on (1) knee angle trajectories[46] at speeds ranging from 0.9 m/s to 2 m/s, (2) heel lift heights[45], (3) overall muscle efficiencies[26], and center of pressure[56]. Parameter variations about the nominal value varied from the maximum (darkest line) to minimum (lightest line). (A-F) Sensitivity of metabolic predictions in the six experiments (nominal result shown by middle curves). Using the maximum parameter value (darkest line) to the minimum value (lightest line) produced variation mostly in magnitude and less in trend. Except for muscle efficiencies, parameter variations led to offsets in magnitudes but not changes in trends.

Table S 1. Comparison of trends, offsets, and goodness-of-fit from curve fitting of the original energy measurements reported in the studied experiments against our model predictions. Both variable efficiency and constant efficiency estimates are shown, parenthetically for the latter. Trend a is in units of W/kg over the experimental variable, which is linear or quadratic depending on the fitting equation. Offset b is in W/kg. Model $\mathbf{R^2}$ values indicate the goodness-of-fit of predictions to fitting equation. Unless reported otherwise, experimental values were derived from the referenced paper (first column). If fits were not reported in the original paper (indicated by †), we performed fits on the empirical data for model comparison. N/A, not available.

**Experimental Parameter (x)**	**Fitting Eqn.**	**Trend a**		**Offset b**		$\mathbf{R^2}$	
		**Model**	**Human**	**Model**	**Human**	**Model**	**Human**
Step Width[19] (m)	ax^2+b	4.848 (6.382)	6.400	2.639 (3.333)	2.19	1.000 (1.000)	0.910
Added Mass[20]: waist (kg)	ax+b	0.012 (0.018)	0.045	2.752 (3.469)	2.36	1.000 (1.000)	0.650
Added Mass[20]: thigh (kg)	ax+b	0.041 (0.054)	0.075	2.752 (3.466)	2.38	1.000 (1.000)	0.720
Added Mass[20]: shank (kg)	ax+b	0.155 (0.179)	0.076	2.752 (3.470)	2.34	1.000 (1.000)	0.610
Added Mass[20]: foot (kg)	ax+b	0.276 (0.298)	0.2	2.743 (3.476)	2.36	1.000 (1.000)	0.850
Extra foot lift[21] (m)	ax+b	7.173 (8.817)	14.43	2.118 (2.710)	1.39	1.000 (1.000)	0.810
Reduced Gravity[22] (g)	ax+b	1.054 (1.646)	0.93	1.078 (1.003)	1.19	1.000 (0.998)	0.550
Flat Walking[23 †] (m/s)	ax+b	4.643 (5.299)	4.299	-1.512 (-1.600)	-0.468	0.988 (0.990)	0.984
Obesity[24 †] (m/s)	ax^2+b	1.273 (1.774)	1.437	0.991 (0.992)	0.77	0.989 (0.995)	0.541
